# Cardiac dysfunction in mixed connective tissue disease: a nationwide observational study

**DOI:** 10.1007/s00296-023-05308-3

**Published:** 2023-03-18

**Authors:** Simon Girmai Berger, Birgit Nomeland Witczak, Silje Reiseter, Thomas Schwartz, Helena Andersson, Siri Opsahl Hetlevik, Kristin Schjander Berntsen, Helga Sanner, Vibke Lilleby, Ragnar Gunnarsson, Øyvind Molberg, Ivar Sjaastad, Mathis Korseberg Stokke

**Affiliations:** 1 Institute for Experimental Medical Research, K. G. Jebsen Center for Cardiac Research, Oslo University Hospital Ullevål, University of Oslo, PB 4956 Nydalen, 0424 Oslo, Norway; 2 Department of Acute Medicine, Oslo University Hospital, Oslo, Norway; 3 Martina Hansens Hospital, Sandvika, Norway; 4 Department of Rheumatology, Oslo University Hospital, Oslo, Norway; 5 Oslo New University College, Oslo, Norway; 6 Department of Medicine, Diakonhjemmet Hospital, Oslo, Norway; 7 Institute for Clinical Medicine, Medical Faculty, University of Oslo, Oslo, Norway; 8 Department of Cardiology, Oslo University Hospital Ullevål, Oslo, Norway; 9 Department of Cardiology, Oslo University Hospital Rikshospitalet, Oslo, Norway

**Keywords:** Connective tissue diseases, Mixed connective tissue disease, Cardiac disease, Echocardiography

## Abstract

We aimed to identify cardiac function in patients with established mixed connective tissue disease (MCTD). This was a cross-sectional case–control study of well-characterised MCTD patients who had previously been included in a nationwide cohort. Assessments comprised protocol transthoracic echocardiography, electrocardiogram and blood samples. In patients only, we evaluated the findings of high-resolution pulmonary computed tomography and disease activity. We assessed 77 MCTD patients (mean age 50.5 ± 12.3 years) with a mean disease duration of 16.4 years, and 59 age- and sex-matched healthy controls (49.9 ± 11.7 years). By echocardiography, measures of left ventricular function, i.e. fractional shortening (38.1 ± 6.4% vs. 42.3 ± 6.6%, p < 0.001), mitral annulus plane systolic excursion (MAPSE) (13.7 ± 2.1 mm vs. 15.3 ± 2.3 mm, p < 0.001) and early diastolic velocity of the mitral annulus (e’) (0.09 ± 0.02 m/s vs. 0.11 ± 0.03 m/s, p = 0.002) were subclinical and lower in patients than controls. Right ventricular dysfunction was found in patients assessed by tricuspid annular plane systolic excursion (TAPSE) (22.7 ± 4.0 mm vs. 25.5 ± 4.0 mm, p < 0.001). While cardiac dysfunction was not associated with pulmonary disease, e’ and TAPSE were found to correlate with disease activity at baseline. In this cohort of MCTD patients, echocardiographic examinations demonstrated a higher frequency of cardiac dysfunction than in matched controls. Cardiac dysfunction was associated with disease activity at baseline, but was independent of cardiovascular risk factors and pulmonary disease. Our study indicates that cardiac dysfunction is part of the multi-organ affliction seen in MCTD.

**Supplementary Information:**

The online version contains supplementary material available at 10.1007/s00296-023-05308-3.

## Introduction

Mixed connective tissue disease (MCTD) is a rare, systemic, autoimmune disease with female predominance, and an estimated population prevalence of 3.8/100,000 in Caucasians [1]. Four different sets of criteria have been developed to classify MCTD for research purposes, with the Alarcón-Segovia criteria as the most widely used. According to these criteria, MCTD is defined according to the presence of circulating anti-U1-RNP auto-antibodies (hereafter anti-RNP), and three or more of the following clinical features: Raynaud’s phenomenon, hand oedema, synovitis, myositis, and arthritis [2].

The clinical features and autoantibody specificities in MCTD overlap partly with other systemic rheumatic diseases, such as systemic lupus erythematosus (SLE) and systemic sclerosis (SSc). However, the disease displays a unique association with human leukocyte antigens, which supports its position as a distinct disease entity [3–7]. MCTD targets multiple organs and may cause different pathologies [8–11]. The pulmonary system is an important example; MCTD predominantly targets the lung parenchyma to cause interstitial lung disease (ILD), but in a subset of patients, it targets small arterioles instead to cause pre-capillary pulmonary arterial hypertension (PAH) [9, 12].

Studies from multiple centres report the involvement of the cardiovascular system in MCTD, and this involvement results in pericarditis, myocarditis, mitral valve prolapse, left ventricular (LV) hypertrophy, LV diastolic dysfunction, conduction abnormalities, or coronary atherosclerosis [10, 11, 13–18]. The prevalence of these features is highly uncertain, ranging from 13 to 65% across referral cohorts [10, 14–17]. In this cross-sectional case–control study, our primary objective was to determine the prevalence and characteristics of cardiac dysfunction in MCTD patients. Our secondary objective was to assess whether or not cardiac dysfunction was a primary manifestation of a stable MCTD phenotype, or consequent to other conditions known to cause cardiac dysfunction, most importantly pulmonary disease.

## Methods

### Patients and controls

This cross-sectional case–control study was performed in 2012–2015 among patients in the previously described Norwegian nationwide MCTD cohort. Baseline evaluation of this cohort, which comprised 147 patients registered with departments of rheumatology at 16 different public hospitals in Norway, was finalised in 2008 [1, 12]. The criteria to be included in the national cohort were: (1) age ≥ 18 years; (2) a positive result in the anti-RNP test; (3) fulfilment of at least one of the following three MCTD criteria sets; Alarcón-Segovia, Kasukawa and modified Sharp’s criteria [2, 3, 19]. Patients with disease features that could be better explained by diagnosis of another connective tissue disorder were excluded.

Both at baseline and at follow-up, we recorded the participants’ medical history and then-current medication, and performed a complete clinical examination as well as lung imaging by high-resolution computed tomography (HRCT). Disease duration was defined as the period from diagnosis to follow-up.

Control subjects were randomly selected from the National Population Register of Norway. Controls were recruited and selected randomly from other studies on rheumatic diseases in which they had also served as controls so that they matched case subjects by age (± 2 years) and sex. Candidates were excluded as controls if they had known cardiovascular disease. All controls who were included gave renewed written consent for this study.

### Definition of disease activity

Since there are no disease activity measures specific for MCTD, we applied measures that had been developed for SLE and SSc as proxies. Hence, disease activity in MCTD was measured according to the SLE disease activity index 2000 (SLEDAI-2 K) and the European League Against Rheumatism scleroderma trial and research group (EUSTAR) activity index [12, 20, 21]. Remission was defined as a SLEDAI-2 K score of zero and a EUSTAR activity index of less than 2.5 [21]. These scores were measured at baseline and at follow-up. Presence of coronary artery disease in patients and controls was defined as previous myocardial infarction or coronary artery bypass surgery.

### Blood samples

Blood samples taken at follow-up were analysed to measure levels of C-reactive protein (CRP), creatine kinase, erythrocyte sedimentation rate (ESR), glycated haemoglobin, high-density lipoprotein (HDL), low-density lipoprotein (LDL), N-terminal pro-B-type natriuretic peptide (NT-pro-BNP) and total cholesterol. Serum levels of anti-RNP autoantibody were measured through the use of the same fully automated fluorescence enzyme immunoassay Phadia 250 (Thermo Fisher Scientific, Phadia GmbH, Freiburg, Germany) in all patients who were included at baseline and at follow-up. Anti-cardiolipin, anti-topoisomerase I (anti-topo I), anti-Smith, anti-Sjögrens Syndrome antigen A (anti-SSA) and antigen B (anti-SSB) were analysed through the use of the same immunoassay.

### Echocardiography

For a full description of the echocardiographic protocol, please see Supplementary Data S1. For our primary objective, cardiac dysfunction was defined as LV systolic dysfunction, LV diastolic dysfunction or RV dysfunction. LV systolic dysfunction was defined as an ejection fraction (LVEF) of ≤ 40%, fractional shortening (FS) of < 25% or mitral annular plane systolic excursion (MAPSE) of < 7 mm [22–24]. LV diastolic dysfunction was defined as the ratio of peak early mitral diastolic velocity (E) to early diastolic mitral annulus velocity (e′), or E/e’, of > 9 or a tricuspid jet maximum velocity (TR_max_) of > 2.8 m/s [22, 25]. RV dysfunction was defined as tricuspid annular plane systolic excursion (TAPSE) of ≤ 17 mm [24]. The echocardiographic probability of pulmonary hypertension was estimated according to the current guidelines: tricuspid regurgitation velocity of > 2.8 m/s or at least two of the following echocardiographic signs suggestive of pulmonary hypertension; TAPSE/systolic pulmonary artery pressure (sPAP) ratio of < 0.55 mm/mmHg, inferior vena cava diameter of > 21 mm with decreased inspiratory collapse (< 50%) or right atrium (RA) area (end-systole) of > 18 cm^2^.

To assess intra- and inter-observer variability, data from a subset of 10 patients were analysed by two observers under the same physical conditions. To assess for intra-observer variability, echocardiographic measurements were measured a second time, 2 weeks after the first.

### Electrocardiogram

The following parameters were collected from a 12-lead electrocardiogram (ECG) at follow-up: rhythm, PR interval, QRS-duration and QT interval, corrected QT interval (QTc), deviations of the ST segment from the isoelectric line and T-wave inversions. LV hypertrophy was defined according to Sokolow–Lyon voltage criteria [26]. ECGs were read by two independent investigators and re-evaluated according to predefined criteria.

### High-resolution computed tomography

High-resolution computed tomography (HRCT) was performed at follow-up as previously described, from which the data have previously been published [12]. The presence and extent of ILD were assessed independently by two chest radiologists. HRCT scans were evaluated in a blinded manner. The presence of ILD was defined as the discovery of ground glass opacities and reticular patterns with or without cysts. In the present study, we classified patients into two groups: ILD positive (+ ILD) and negative (−ILD).

### Statistical analysis

Data are presented as mean values with standard deviation (SD) and categorical variables as absolute frequencies. In continuous, normally distributed variables, differences between groups were examined using the independent-sample Student’s t-test. For categorical variables, the Chi-squared test was used to compare the groups. Pearson's correlation analysis was used to explore selected echocardiographic variables. Intra- and inter-observer variabilities were expressed as the mean absolute difference between the observers ± SD and as percentages of their mean. We calculated the intra-class correlation coefficients (ICCs) respectively. An ICC of > 0.9 was considered to show excellent reliability; 0.9 > ICC > 0.75 good reliability, 0.75 > ICC > 0.5 moderate reliability and an ICC of ≤ 0.5 indicated poor reliability. All tests were two-sided, and a p-value of < 0.05 was considered statistically significant. All patients with incomplete data were removed from the analysis. Data analysis was performed through the use of SPSS software, version 22 (IBM SPSS, Armonk, NY, USA).

## Results

### Population characteristics

**Fig. 1:**
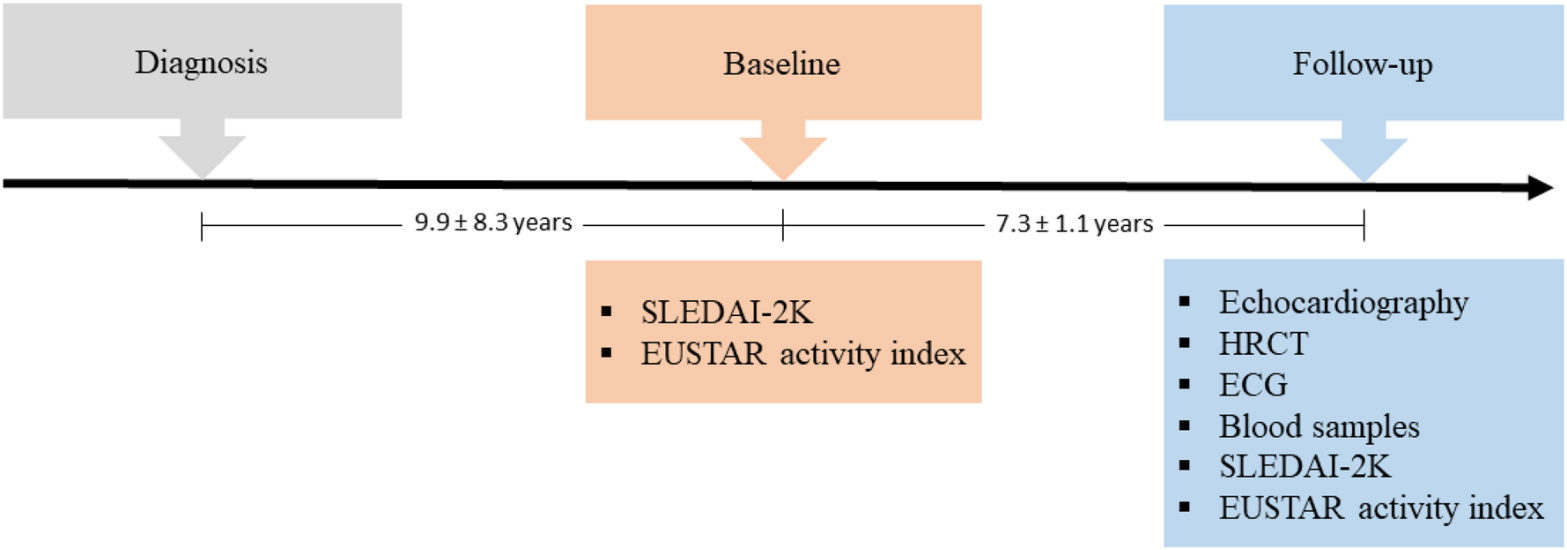
Study outline showing patient examinations that were performed at baseline (establishment of MCTD cohort) and at follow-up. Disease activity was measured at baseline and at follow-up, when echocardiography and HRCT were performed and blood samples acquired

Seventy-seven patients with established MCTD were included from the Norwegian nationwide MCTD cohort for the present follow-up study. Of these, 60 (78%) were female (Table 1) [1, 12]. Patients were diagnosed with MCTD at a mean age of 34.0 ± 12.7 years, and were included in the cohort and examined at baseline at a mean age of 43.9 ± 12.2 years (Fig. 1). Their mean age at the time of the present follow-up study was 50.5 ± 12.3 years (range 25–81 years) and they had a mean disease duration of 16.4 ± 8.3 years (Fig. 1). Three patients included in the follow-up study did not have data available from baseline examinations. From our available cohorts of healthy controls, we identified 59 people who could be age- and sex-matched to the MCTD patients. These controls had a mean age of 49.9 ± 11.7 years, and 47 of them were female (80%).

**Table 1:** Demographic and clinical characteristics in patients with mixed connective tissue disease included in follow-up study

	n	MCTD total ± SD
Characteristics		
Female sex, n (%)	77	60 (78)
Age at disease onset, years	74	30.7 ± 12.2
Age at diagnosis, years	74	34.0 ± 12.7
Age at baseline examination, years	74	43.9 ± 12.2
Age at follow-up examination, years	77	50.5 ± 12.3
Disease duration, years	74	16.4 ± 8.3
Fulfilled Alarcón-Segovia and Villarreal criteria, n (%)	72	63 (88)
Fulfilled Kasukawa et al. criteria, n (%)	71	60 (85)
Fulfilled modified Sharp criteria, n (%)	72	68 (94)
Arthritis, n (%)	74	9 (12)
Pericarditis, n (%)	74	1 (1)
Puffy hands, n (%)	74	36 (49)
Raynaud’s phenomenon, n (%)	74	68 (92)
Sclerodactyly, n (%)	72	21 (29)
Oesophageal dilatation on x-ray, n (%)	71	30 (42)
Myositis, n (%)	74	1 (1)
Current medication:		
Azathioprine, n (%)	72	13 (18)
Corticosteroids, n (%)	73	35 (47)
Daily corticosteroid dose, mg	33	5.6 ± 3.9
Methotrexate, n (%)	73	13 (18)
NSAIDs, n (%)	75	21 (28)
Hydroxychloroquine, n (%)	73	36 (49)
Mycophenolate, n (%)	73	1 (3)
Monotherapy, n (%)^a^	73	28 (36)
Combination therapy, n (%)^a^	73	9 (12)
No medication, n (%)^a^	73	40 (52)
Disease activity scores at follow-up:		
EUSTAR activity index	74	0.5 ± 0.7
SLEDAI-2 K score	74	1.4 ± 2.0
Disease activity scores at baseline:		
EUSTAR activity index	74	0.5 ± 0.8
SLEDAI-2 K score	74	2.5 ± 2.5

Values are mean ± SD. All parameters from the time of the follow-up study unless otherwise stated

^a^Therapy includes corticosteroids at ≤ 5 mg/day, azathioprine, mycophenolate and/or methotrexate

Regarding MCTD criteria, 63 of the 77 patients (88%) fulfilled the criteria of Alarcón-Segovia and Villareal [2], 60 (85%) fulfilled the criteria set by Kasukawa et al. [19], and 68 (94%) met the modified Sharp’s criteria for MCTD [27]. The mean anti-RNP titre at follow-up was 83.8 ± 93.2 × 10^−3^ U/l. None of the MCTD patients had findings better explained by another systemic connective tissue disease. At follow-up, no patient tested positive for anti-topo I. Two patients were weakly positive for anti-Sm. Serum anti-SSA antibodies were present in 21% of the group (16 of 77 patients) and anti-SSB antibodies in 5% (four of 77). At follow-up, we found that three patients had shifted to SLE, two to RA, one to SSc and one patient had shifted to anti-Jo1 positive anti-synthetase syndrome.

Disease activity according to the total SLEDAI-2 K score had decreased from the baseline 2.5 ± 2.5 to 1.4 ± 2.0 at follow-up, while the EUSTAR activity index remained unchanged between the two time points, 0.5 ± 0.8 at baseline vs. 0.5 ± 0.7 at follow-up. At an individual level, the EUSTAR-score increased by 1.2 ± 0.4 in 15 patients and decreased by 1.2 ± 0.7 in 13 patients. The number of patients in remission, defined as showing a SLEDAI-2 K score = 0 and a EUSTAR activity index of < 2.5, increased from 25 (34%) at baseline to 35 (47%) at follow-up. At follow-up, 37 patients (48%) were being treated with immunosuppressive drugs; these included corticosteroids (47%), methotrexate (18%), azathioprine (18%), and mycophenolate (3%). Twenty-eight patients (36%) received monotherapy, nine (12%) combination therapy, and 40 (52%) received no medical therapy.

### Cardiovascular risk factors in patients and controls

Cardiovascular risk factors and levels of biomarkers for cardiovascular disease that were measured at follow-up are summarised in Table 2. More patients with MCTD than controls were smokers (50% vs. 27%; p = 0.01). The patients showed lower plasma concentrations of HDL (p < 0.001) than did the controls, but there were no significant differences in levels of total cholesterol or LDL, or in the HDL/LDL ratio. MCTD patients had higher NT-pro-BNP levels (p = 0.01), although the values for both groups were low. Compared with controls, patients with MCTD had higher serum levels of high-sensitivity CRP (p = 0.04) and ESR (p < 0.001). Five patients had histories of myocardial infarction, and four others had histories of cerebrovascular disease.

**Table 2:** Cardiovascular risk factors in patients with mixed connective tissue disease at follow-up, and in controls

	n	MCTD total	n	Controls	p-value
Characteristics					
Age at examination, years	77	50.5 ± 12.3	59	49.9 ± 11.7	0.76^a^
Female sex, n (%)	77	60 (78)	59	47 (80)	0.81^b^
Height, cm	77	169 ± 9	57	170 ± 8	0.64^a^
Weight, kg	77	73.0 ± 16.9	58	71.6 ± 11.2	0.57^a^
Body mass index, kg/m^2^	77	25.3 ± 4.8	57	24.6 ± 2.7	0.35^a^
BP systolic, mmHg	76	125 ± 18	56	128 ± 17	0.25^a^
BP diastolic, mmHg	76	73 ± 9	56	76 ± 11	0.07^a^
HR, beats/min	75	66 ± 11	59	65 ± 11	0.79^a^
Smokers, daily, n (%)	72	36 (50)	45	10 (27)	0.01^b^
LDL cholesterol, mmol/l	74	2.9 ± 0.9	19	3.0 ± 0.6	0.72^a^
HDL cholesterol, mmol/l	74	1.5 ± 0.5	19	1.9 ± 0.4	< 0.001^a^
HDL/LDL ratio	74	0.6 ± 0.3	19	0.7 ± 0.3	0.09^a^
Total cholesterol, mmol/l	74	4.7 ± 1.1	10	4.6 ± 1.2	0.77^a^
NT-pro-BNP, ng/l	73	14.9 ± 17.0	44	8.05 ± 6.24	0.01^a^
Creatine kinase, U/l	75	121 ± 146	57	135 ± 118	0.56^a^
HbA1c, %	71	5.6 ± 0.5	12	5.2 ± 0.3	0.02^a^
CRP, mg/l	75	3.8 ± 8.1	55	1.4 ± 1.9	0.04^a^
ESR, mm/h	77	13.7 ± 12.7	57	6.0 ± 3.9	< 0.001^a^
Anti-RNP, × 10^−3^ U/l	74	83.8 ± 93.2	NA	NA	NA
Current medication					
Ca^2+^ antagonists, n (%)	77	14 (18)	59	1 (2)	0.002^b^
β adrenoceptor blockers, n (%)	77	4 (5)	59	2 (3)	0.61^b^
Statins, n (%)	77	4 (5)	59	7 (12)	0.16^b^
Angiotensin-II- receptor blockers, n (%)	77	6 (8)	59	2 (3)	0.28^b^
Vitamin K antagonists, n (%)	77	1 (1)	59	0 (0)	0.38^b^
NSAIDs, n (%)	77	8 (10)	59	2 (3)	0.12^b^
Nitrates, n (%)	77	1 (1)	59	0 (0)	0.38^b^
Diuretics, n (%)	77	1 (1)	59	1 (2)	0.85^b^
I_1_- Imidazoline receptor, n (%)	77	0 (0)	59	1 (2)	0.25^b^
P2Y-inhibitors, n (%)	77	0 (0)	59	1 (2)	0.25^b^

Values are mean ± SD

*BP* blood pressure, *Ca*^*2+*^ calcium, *HbA1c* glycated haemoglobin, *HDL* high-density lipoprotein, *HR* heart rate, *LDL* low-density lipoprotein, *NSAIDs* non-steroidal anti-inflammatory drugs, *NT-pro-BNP* N-terminal pro-BNP

^a^Student’s t-test

^b^χ^2^ test

### Electrocardiological characteristics in patients and controls

Standard ECG intervals in patients with MCTD and the controls at follow-up are shown in Table 3. ECG abnormalities were detected in 15 patients (19%) compared with five controls (11%) (p = 0.21). These ECG abnormalities comprised atrioventricular block (three in patients, two in controls), LV hypertrophy (four vs. two), right bundle branch block (two vs. zero), and T-wave inversions (seven vs. one). Supraventricular premature complexes were recorded in four patients and no controls.

**Table 3:** Electrocardiographic and echocardiographic characteristics in patients with mixed connective tissue disease at follow-up, and in controls

Variables	n	MCTD total	n	Controls	p-value
ECG					
P, ms	76	88 ± 16	46	94 ± 12	0.03^a^
PR, ms	76	165 ± 25	46	169 ± 24	0.39^a^
QRS, ms	77	85 ± 16	46	83 ± 12	0.56^a^
QTc, ms	77	398 ± 32	46	392 ± 25	0.25^a^
Supraventricular extra systole, n (%)	77	4 (5)	46	0 (0)	0.12^b^
Pathological ECG, n (%)	77	15 (19)	46	5 (11)	0.21^b^
Echocardiography					
B-mode					
LV diastolic volume, cm^3^	72	85.2 ± 26.5	59	89.3 ± 23.0	0.36^a^
LV systolic volume, cm^3^	72	35.9 ± 15.2	59	36.0 ± 12.6	0.97^a^
LV ejection fraction, %	72	58.7 ± 8.1	59	60.1 ± 8.6	0.35^a^
LA area, cm^2^	76	16.9 ± 3.7	59	16.4 ± 3.8	0.48^a^
RA area, cm^2^	76	15.3 ± 3.5	59	15.4 ± 3.2	0.80^a^
M-mode					
IVS diastole, cm	75	1.0 ± 0.2	56	1.0 ± 0.2	0.53^a^
IVS systole, cm	75	1.3 ± 0.2	58	1.4 ± 0.2	0.09^a^
LVID diastole, cm	75	4.8 ± 0.5	59	4.9 ± 0.4	0.07^a^
LVID systole, cm	75	3.0 ± 0.5	55	2.9 ± 0.4	0.21^a^
Fractional shortening, %	75	38.1 ± 6.4	59	42.3 ± 6.6	< 0.001^a^
PW diastole, cm	75	0.9 ± 0.2	59	0.9 ± 0.1	0.01^a^
PW systole, cm	74	1.5 ± 0.3	59	1.5 ± 0.2	0.20^a^
MAPSE, mm	75	13.7 ± 2.1	59	15.3 ± 2.3	< 0.001^a^
TAPSE, mm	73	22.7 ± 4.0	58	25.5 ± 4.0	< 0.001^a^
Doppler					
MV E velocity, m/s	76	0.73 ± 0.15	59	0.74 ± 0.14	0.67^a^
MV A velocity, m/s	75	0.61 ± 0.20	59	0.58 ± 0.17	0.39^a^
MV E/A ratio	75	1.28 ± 0.38	59	1.36 ± 0.41	0.25^a^
LVOT stroke volume, ml	72	49.3 ± 14.5	59	53.3 ± 15.0	0.13^a^
LVOT cardiac output, l/min	72	3.2 ± 1.0	59	3.4 ± 1.0	0.27^a^
TV_max_, m/s	74	0.46 ± 0.08	59	0.49 ± 0.10	0.08^a^
TR_max_, m/s	76	1.76 ± 0.55	58	1.54 ± 0.58	0.03^a^
sPAP, mmHg	76	13.9 ± 7.5	58	10.6 ± 7.1	0.01^a^
AV_max_, m/s	74	1.24 ± 0.27	59	1.19 ± 0.33	0.33^a^
PV_max_, m/s	75	0.70 ± 0.15	59	0.77 ± 0.16	0.01^a^
Tissue doppler					
e’ velocity, m/s	76	0.09 ± 0.02	58	0.11 ± 0.03	0.002^a^
E/e’ ratio	76	8.07 ± 2.46	58	7.22 ± 2.39	0.049^a^
Strain					
LV global longitudinal strain, %	75	22.4 ± 3.0	57	23.0 ± 2.5	0.26^a^
LV global circumferential strain, %	74	27.2 ± 4.3	55	27.0 ± 3.3	0.74^a^
RV basal diameter, cm	71	3.4 ± 0.5	58	3.6 ± 0.5	0.07^a^
RVOT proximal diameter, cm	64	2.9 ± 0.4	54	3.1 ± 0.5	0.02^a^
RV longitudinal diameter, cm	57	6.9 ± 0.7	37	7.1 ± 0.7	0.22^a^
Right ventricular systolic pressure, mmHg	57	19.0 ± 7.9	40	16.4 ± 7.2	0.10^a^

Values are mean ± SD

*AV* aortic valve, *e’* early diastolic mitral annulus velocity, *IVS* intraventricular septum, *LA* left atrium, *LV* left ventricle/ventricular, *LVID* left ventricular internal diameter, *MAPSE* mitral annular plane systolic excursion, *MV* mitral valve, *MV A* MV A-wave, *MV E* MV E-wave, *P* P-wave, *PR* PR interval, *PV* pulmonary vein, *PW* posterior wall, *QRS* QRS complex, *QTc* corrected QT interval, *RA* right atrium, *RV* right ventricle/ventricular, *sPAP* systolic pulmonary artery pressure, *TAPSE* tricuspid annular plane systolic displacement, *TR* tricuspid regurgitation

^a^Student’s t-test

^b^χ^2^ test

### Echocardiographic characterisation of patients and controls

Echocardiographic characteristics at follow-up are presented in Table 3. LV systolic dysfunction was present in three of 76 patients (4%); LV diastolic dysfunction was detected in 25 of 76 patients (33%), and RV dysfunction, defined as TAPSE of ≤ 17 mm, was present in eight of 73 patients (11%). A total of 30 patients (39%) showed evidence of cardiac dysfunction, which was defined as LV systolic, LV diastolic or RV dysfunction, compared with 13 controls (22%) (p = 0.04) (Supplementary Table S1). The probability of pulmonary hypertension as measured by echocardiogram was found to be elevated in two of 77 patients (2.6%). Conventional measurements of cardiac dimensions and volumes were within normal limits, and no significant differences were detected in these measures between patients with MCTD and controls.

To assess LV systolic function, we measured FS, MAPSE, EF and GLS. FS (38.1 ± 6.4% vs. 42.3 ± 6.6%, p < 0.001) and MAPSE (13.7 ± 2.1 mm vs. 15.3 ± 2.3 mm, p < 0.001) were found to be lower in patients than in controls (Table 3). Mean LVEF and global longitudinal strain were within the normal ranges and did not significantly differ between patients and controls. LV diastolic function as measured by e’ was lower in MCTD patients than controls (0.09 ± 0.02 m/s vs. 0.11 ± 0.03 m/s, p = 0.002), and the E/e’ ratio was higher (8.07 ± 2.46 vs. 7.2 ± 2.39, p = 0.049) (Table 3). The mitral E-wave/A-wave ratio was not significantly different between patients and controls. Levels of NT pro-BNP were correlated with LV dysfunction (r_sp_ = 0.42, p < 0.001). In patients with LV dysfunction, the amount of NT pro-BNP was increased compared with patients without LV dysfunction (24.5 ± 23.0 ng/l vs. 9.5 ± 9.4 ng/l, p < 0.001). E/e’ was correlated to levels of NT-pro-BNP in the serum of patients with MCTD (r_sp_ = 0.42, p < 0.001). RV systolic function, as assessed by TAPSE, was reduced in patients with MCTD compared to controls (22.7 ± 4.0 mm vs. 25.5 ± 4.0 mm, p < 0.001) (Table 3). TAPSE was found to be inversely correlated to levels of serum NT-pro-BNP in patients with MCTD (r_sp_ = − 0.28, p = 0.02). Echocardiographic parameters were analysed to estimate right-sided pressures and RV function (Table 3). TR_max_ was found to be higher in patients than in controls (1.76 ± 0.55 m/s vs. 1.54 ± 0.58 m/s, p = 0.03). There was no significant difference in estimated RV systolic pressure between patients and controls. Intra- and inter-observer variabilities are summarised in Supplementary Table S2. Mean differences were not significantly different and were < 10%, and reproducibility ranged between good and excellent.

### Association between cardiac function and disease activity in patients with MCTD

**Fig. 2:**
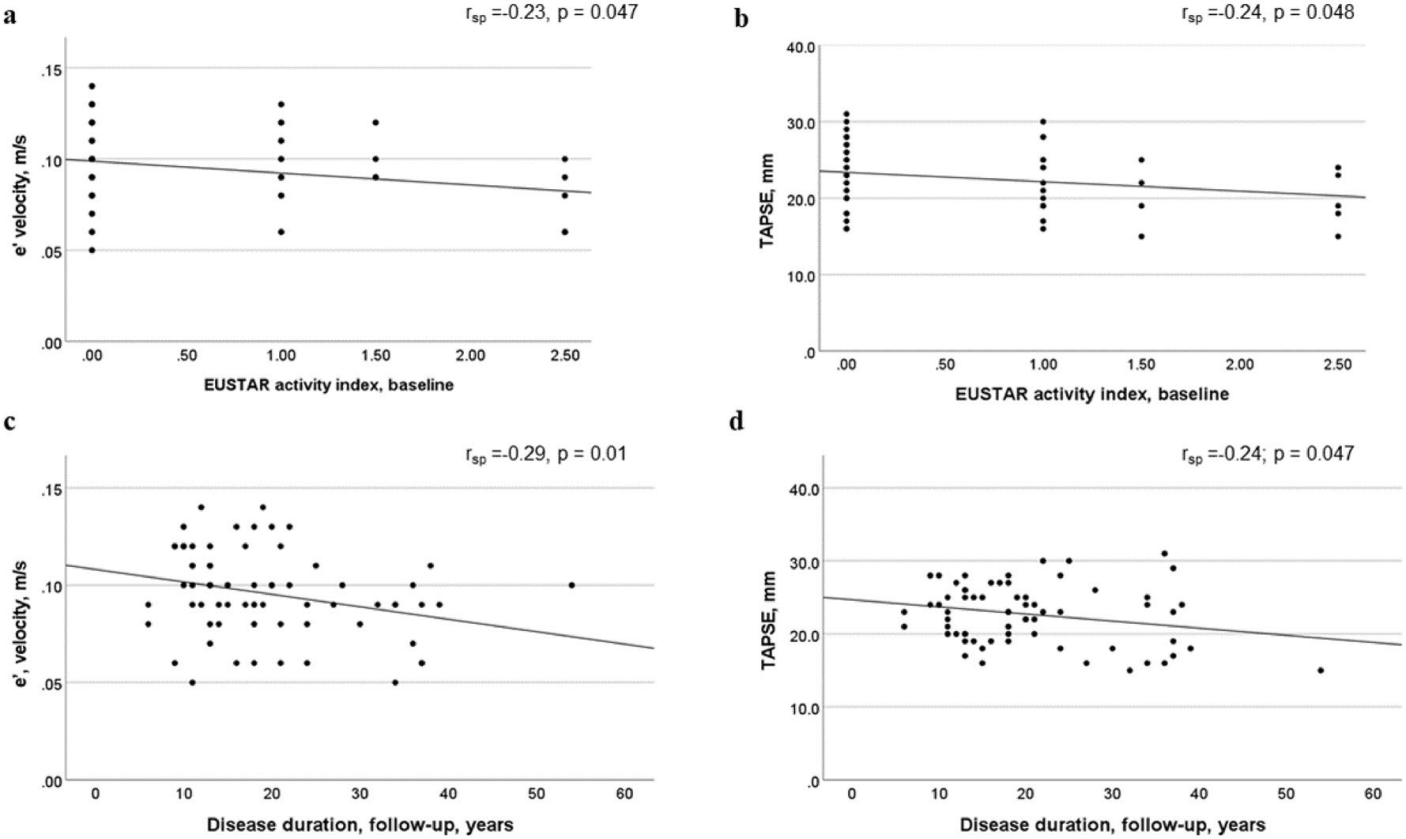
Correlations between cardiac function and disease activity at baseline, and between cardiac function and disease duration at follow-up: **a** and **b** diastolic function (e’ velocity, in m/s) at follow-up and RV function (TAPSE, in mm) at follow-up, both related to the patients’ EUSTAR activity indices at baseline; **c** and **d** diastolic function (e’ velocity, in m/s) and RV function (TAPSE, in mm) related to disease duration at follow-up, in years. *r*_*sp*_ Spearman correlation coefficient

To examine the predictive value of early disease activity, we analysed the association between the EUSTAR and SLEDAI-2 K activity indices at baseline and markers of LV and RV dysfunction at follow-up. The EUSTAR index at baseline was inversely correlated with e’ (r_sp_ = − 0.23, p < 0.05) and TAPSE (r_sp_ = − 0.24, p < 0.05) at follow-up (Fig. 2a, b). Levels of sclerodactyly in patients at baseline were correlated with lower e’ (r_sp_ = − 0.33, p = 0.004) and E/e’ (r_sp_ = 0.40, p < 0.001) at follow-up. Increased SLEDAI-2 K scores at baseline were inversely correlated with e’ at follow-up (r_sp_ = − 0.27, p = 0.02); however, no correlation was found between SLEDAI-2 K and TAPSE. Additionally, MCTD patients with active disease compared with patients in remission (defined as having a SLEDAI-2 K score of zero and a EUSTAR activity index of < 2.5) at baseline had lower TAPSE measures (2.20 ± 0.34 mm vs. 2.43 ± 0.41 mm, p = 0.02) and e’ (0.09 ± 0.02 m/s vs. 0.11 ± 0.02 m/s, p = 0.03) at follow-up.

At follow-up, cardiac dysfunction was associated with proximal muscle weakness (p < 0.01), but no correlation was found with smoking status or immunosuppressant or medication use. Six out of seven patients who tested positive for anti-cardiolipin had cardiac dysfunction (p = 0.01). A borderline correlation was demonstrated between TAPSE and disease duration in patients (r_sp_ = − 0.24, p < 0.05), while no correlation was found with age (Fig. 2d). It was also found that e’ was inversely correlated with both disease duration (r_sp_ = − 0.29, p = 0.01) and age (r_sp_ = − 0.68, p < 0.001) (Fig. 2c).

### Cardiac function in patients with MCTD according to presence of ILD

A possible causal explanation for cardiac dysfunction in patients with MCTD is pulmonary disease. Therefore, we analysed the data from the patients according to evidence of ILD by HRCT (Supplementary Table S3). ILD was found in 22 patients (29%). These patients were older at disease onset than were those patients without ILD (36.0 ± 13.9 years vs. 29.2 ± 10.7 years, p = 0.03). However, age, disease duration and the medication being taken at follow-up were not significantly different. Patients with ILD had lower levels of HDL cholesterol (1.3 ± 0.4 mmol/l vs. 1.6 ± 0.4 mmol/l, p = 0.04), while no other cardiovascular risk factors were found to differ between the patients with and without ILD.

There were no significant differences in ECGs between the MCTD patients who had and did not have ILD (Supplementary Table S4). The RV systolic pressure was higher in patients with ILD (22.5 ± 6.9 mmHg vs. 17.7 ± 8.2 mmHg, p = 0.04), but TAPSE and e’ were not significantly different between patients with and without ILD. Furthermore, only one of eight patients with reduced measures of TAPSE had ILD.

## Discussion

We studied cardiac function as assessed by echocardiography in patients who had MCTD from a Norwegian nationwide cohort. Our main finding was evidence of cardiac dysfunction in these patients, as shown by lower group means for the key functional parameters FS, MAPSE, e` and TAPSE, and a higher prevalence of echocardiographic functional parameters that were below reference values in patients than were found in the controls. Cardiac dysfunction at the time of our follow-up study was associated with disease activity at the time of baseline inclusion in the registry, but was independent of cardiovascular risk factors and pulmonary disease. These data suggest that primary cardiac dysfunction can be a part of MCTD. This finding underlines the importance of targeted and repeated cardiac evaluation in these patients.

We found evidence of impaired systolic function in the hearts of the MCTD cohort compared with the controls. Mean FS and MAPSE measures were lower in patients than they were in the controls, but within the normal range. However, manifest LV systolic dysfunction, as measured by systolic parameters below reference values, was found in only 4% of the patients.

Our finding of normal LVEF in patients with MCTD is in line with results reported in previous publications [15, 16]. Although LVEF is the most widely used marker of LV systolic function, its dominant position for this purpose has been a matter of debate for years [28]. It is therefore noteworthy that MAPSE, a sensitive and robust early marker of LV systolic function, was affected in our patients with MCTD [23]. MAPSE is easily measured, and a well-established prognostic marker in patients with heart failure [29]. Impaired MAPSE in patients with MCTD has been reported in similar studies of patients with juvenile-onset MCTD [30].

It is possible that we found signs of dysfunction according to measures of MAPSE but not those of LVEF because our MCTD cohort had modest reductions in LV function.

LV diastolic function as assessed by e’ was lower in patients compared with controls. By itself, e’ should be treated with caution as a parameter of diastolic function. However, consideration of all diastolic parameters showed a higher prevalence of measurements outside reference values in the patients than in the controls.

In our patients, the EUSTAR activity indices at baseline and disease duration were found to be associated with lower e’ measures. This finding indicates that impaired LV relaxation in MCTD is related to the severity of the disease at baseline and cumulative disease load, which may include inflammatory load, disease damage and disease progression.

Interestingly, EUSTAR activity showed a stronger correlation with cardiac dysfunction than did SLEDAI-2 K. This indicates that a phenotype of SSc-like disease in MCTD patients is more likely to be associated with cardiac dysfunction than is a phenotype of SLE-like disease. One could speculate that the same underlying mechanisms of diffuse fibrosis and vascular abnormalities that affect the skin and lungs may cause diastolic impairment in the heart.

The mean TAPSE was reduced in patients with MCTD compared with the controls, and 11% of the patients in our cohort were found to have impaired RV function, defined as a TAPSE of ≤ 17 mm [24]. Importantly, this was independent of the presence of ILD.

TAPSE is a variable that is widely used to assess RV systolic function, as recommended by current guidelines [24]. The most likely cause of RV dysfunction in patients with MCTD is pulmonary hypertension, primary or secondary to pulmonary disease [31].

Indeed, PAH remains the major cause of death in MCTD [11], and Vegh et al. have demonstrated that MCTD patients with PAH have both RV and LV diastolic dysfunction [16]. It has been shown that TAPSE can be used as a reliable predictor of survival in patients with PAH [32]. In our MCTD cohort, patients with ILD had higher mean RV systolic pressures than the controls. However, only one of eight (13%) MCTD patients who had a reduced TAPSE had ILD. Additionally, RV systolic pressure did not differ significantly between patients and controls. Therefore, the reduced RV function that was found in this cohort of patients with MCTD did not seem to be secondary to pulmonary disease.

In our study, TAPSE was found to be correlated with EUSTAR activity index at baseline and with disease duration for MCTD. Additionally, MCTD patients with active disease at baseline (defined as a SLEDAI-2 K score of > 0 and a EUSTAR activity index of > 2.5) showed lower TAPSEs than MCTD patients in remission at baseline. We speculate that the decline in RV function in MCTD patients over time was related to disease progression, either as chronic inflammation or as organ-specific cardiac effect and/or as part of general organ remodelling.

Chronic inflammation is pivotal in the development of myocardial disease and atherosclerosis [33]. In patients with MCTD, endothelial dysfunction may be accelerated due to chronic systemic inflammation that promotes cardiovascular disease [33]. In this study, patients with MCTD displayed elevated levels of inflammatory markers such as CRP and ESR, compared with the controls. Furthermore, the levels of these inflammatory markers were higher in patients with ILD than in those without ILD; however, cardiac function did not differ significantly between these two patient groups.

Regardless of the mechanisms that underlie cardiac dysfunction, the finding that TAPSE was reduced in patients with MCTD independent of the presence or absence of ILD should be explored further to identify possible mechanisms. TAPSE might have a prognostic value in patients with MCTD independent of its association with pulmonary disease. This finding should also be confirmed in prospective studies of patients with MCTD.

### Limitations

We have echocardiographic data from one time point only. Future prospective studies with serial cardiac examinations should be performed. Additionally, TAPSE was used as the sole diagnostic criterion for RV dysfunction. Future studies should include the evaluation of RV function through the use of complementary imaging methods, including cardiac magnetic resonance imaging, at different time points to further characterise LV and RV structure and function and their association with the course of disease. Additionally, although our data do not indicate an association between ILD and cardiac dysfunction in patients with MCTD, a larger cohort of patients who have been examined by right-sided heart catheterisation is required to evaluate fully this possible association.

## Conclusion

We found that 39% of patients in our nationwide Norwegian cohort of patients with MCTD had evidence of cardiac dysfunction that affected one or both ventricles, compared with 22% of age- and sex-matched controls. Cardiac dysfunction at follow-up was associated with disease activity at baseline, independent of the presence of pulmonary disease. This indicates that cardiac dysfunction could be a primary manifestation of MCTD. Systematic follow-up of cardiac function should be performed in patients with MCTD, and future studies should explore the mechanisms that underlie cardiac dysfunction in these patients.

## Supplementary Information

Below is the link to the electronic supplementary material.

Supplementary file1 (DOCX 27 KB)

## Data Availability

The data that underlie this article cannot be shared publicly in order to ensure the privacy of the individuals who participated in the study, and due to legal restrictions regarding the sharing of sensitive data.
