# Supplementary material for: Cardiac dysfunction in mixed connective tissue disease: a nationwide observational study
Source: Rheumatol Int. 2023 Mar 18;43(6):1055–65. doi: 10.1007/s00296-023-05308-3 (PMC10126085; doi:10.1007/s00296-023-05308-3)
Supplement: Supplementary file 1 — Supplementary file1 (DOCX 27 KB) [file 296_2023_5308_MOESM1_ESM.docx]

# Supplementary Data S1

### Echocardiography

Two-dimensional (B-mode), M-mode, Doppler, strain and Doppler tissue imaging echocardiography were performed at follow-up with Vivid 7 and Vivid E9 ultrasound machines (GE Medical Systems, Milwaukee, Wisconsin^®^, USA). All images were acquired at rest during spontaneous breathing by a cardiologist unaware of the subject’s clinical condition. A minimum of three cycles were recorded per image. All echocardiographic measurements were performed by a single investigator.

The left atrial (LA) and right atrial (RA) area were quantified at end-systole using the apical 4-chamber B-mode view. LV diameter, wall thicknesses, and fractional shortening (FS) were measured in M-mode. LV volumes and LV ejection fraction (LVEF) were calculated from apical 2- and 4-chamber views. M-mode images were obtained by placing the M-mode cursor at the lateral and septal angles of the mitral annulus in the apical 4-chamber view, and average mitral annular plane systolic excursion (MAPSE) was calculated.

Myocardial strain was quantified from LV apical 2-chamber, 4-chamber and short-axis view. Images were analyzed using a semi-automated 2D speckle-tracking software, (EchoPac PC, version 202, GE Healthcare, Horten, Norway). The LV endocardial borders were traced manually in the end-diastolic frame. The software generated a region of interest (ROI) between endocardial and epicardial layers that was manually adjusted to fit the myocardial thickness. Tracking through the cardiac cycle was manually confirmed. The LV was divided into six segments and each segment's longitudinal strain was measured. The software calculated the global longitudinal strain (GLS) by averaging the local longitudinal strains of the LV*.*

Indices of LV diastolic function, peak early (E) and its deceleration time, and peak late (A) mitral diastolic velocities were obtained by pulsed-wave Doppler (PW). Diastolic function was also assessed using Doppler tissue imaging (DTI), enabling measurements of the peak early diastolic velocity, e’, at the lateral and septal aspects of the mitral annulus. The lateral and septal e' were averaged, and E/e' was calculated.

RV dimensions were measured as recommended (1). RV basal and longitudinal diameters were measured from the apical view at end-diastole while RV proximal diameter from the parasternal long-axis view. Tricuspid annular plane systolic excursion (TAPSE) was measured in M-mode as the difference in the displacement of the RV base from end-diastole to end-systole.

RV systolic pressure was estimated by measuring TR_max_ by continuous wave (CW) spectral Doppler using modified Bernoulli equation ΔP = 4 × V_max_^2^ and adding an estimated right atrial pressure (RAP). RAP was estimated by assessing the inferior vena cava (IVC) size and collapsibility during inspiration.

Peak aortic valve velocity (AV_max_) and peak pulmonary valve velocity (PV_max_) were measured using continuous-wave Doppler. Valvular regurgitation >grade 1 was considered significant.

# Supplementary Table S1

| Supplementary Table S1. Cardiac dysfunction in patients with mixed connective tissue disease at follow-up. | | | | |
| --- | --- | --- | --- | --- |
| Variables | Cut-off | MCTD, n (%) | Controls, n (%) | p-value |
| LV systolic function |  | 3 (4) | 1 (2) | 0.44 ^a^ |
| LV ejection fraction, % | ≤ 40 % | 1 (1) | 1 (1) | 0.88^a^ |
| Fractional shortening, % | <25 % | 2 (3) | 0 (0) | 0.21^a^ |
| MAPSE, mm | < 7 mm | 0 (0) | 0 (0) | NA |
| Diastolic function |  | 25 (33) | 11 (19) | 0.07 ^a^ |
| E/e’ ratio | > 9 | 24 (32) | 11 (19) | 0.1^a^ |
| TR_max_, m/s | > 2.8 m/s | 1 (1) | 0 (0) | 0.38^a^ |
| RV function (TAPSE, mm) | ≤ 17 mm | 8 (11) | 1 (2) | 0.04^a^ |
| Cardiac dysfunction^a^ |  | 30 (39) | 13 (22) | 0.04^a^ |
| ^a^Cardiac dysfunction defined as the presence of LV or RV dysfunction. MAPSE, mitral annular plane systolic excursion; RV, right ventricle/ventricular; TAPSE, tricuspid annular plane systolic displacement; TR, tricuspid regurgitation; MV E, MV E-wave; ^a^X^2^ test | | | | |

# Supplementary Table S2

| Supplementary Table S2. Intra- and inter-observer variabilities of echocardiographic measurements | | | | | | |
| --- | --- | --- | --- | --- | --- | --- |
|  | Intraobserver | | | Interoberver | | |
| Variables | Mean absolute difference | Mean absolute, % | ICC | Mean difference ± SD | Mean absolute, % | ICC |
| LV systolic function |  |  |  |  |  |  |
| LVEF% | 3.1 ± 2.62 | 5.6 ± 4.6 | 0.84 | 3.3 ± 1.68 | 6.3 ± 3.4 | 0.87 |
| FS % | 1.8 ± 0.9 | 5.1 ± 2.5 | 0.95 | 2.3 ± 3.4 | 6.7 ± 5.1 | 0.87 |
| MAPSE, mm | 0.06 ± 0.07 | 4.5 ± 5.4 | 0.90 | 0.07 ± 0.06 | 5.1 ± 3.7 | 0.90 |
| Diastolic function |  |  |  |  |  |  |
| MV E velocity, m/s | 0.03 ± 0.04 | 3.2 ± 4.0 | 0.99 | 0.04 ± 0.03 | 4.7 ± 3.1 | 0.99 |
| MV A velocity, m/s | 0.03 ± 0.03 | 4.4 ± 4.2 | 0.99 | 0.02 ± 0.04 | 3.2 ± 6.4 | 0.99 |
| e’ velocity, m/s | 0.00 ± 0.00 | 0.6 ± 3.1 | 0.99 | 0.01 ± 0.01 | 6.3 ± 6.6 | 0.95 |
| TR_max_, m/s | 0.03 ± 0.05 | 1.9 ± 3.3 | 0.99 | 0.05 ± 0.05 | 3.5 ± 3.9 | 0.99 |
| sPAP mmHg | 0.05 ± 0.79 | 0.6 ± 5.6 | 0.99 | 0.97 ± 0.98 | 6.9 ± 5.3 | 0.99 |
| RV function (TAPSE, mm) | 0.08 ± 0.06 | 3.7 ± 2.8 | 0.99 | 0.12 ± 0.1 | 5.6 ± 4.4 | 0.97 |
| Values are absolute mean ± SD. ICC, Intraclass Correlation Coefficient; MAPSE, mitral annular plane systolic excursion; LVEF, left ventricular ejection fraction; MV, mitral valve; MV A, MV A-wave; MV E, MV E-wave; RV, right ventricle/ventricular; sPAP, systolic pulmonary artery pressure; TAPSE, tricuspid annular plane systolic displacement; TR, tricuspid regurgitation; | | | | | | |

# Supplementary Table S3

| Supplementary Table S3. Cardiovascular risk factors in patients with mixed connective tissue disease at follow-up, and in controls. | | | | | |
| --- | --- | --- | --- | --- | --- |
| Characteristics | n | Patients -ILD | n | Patients +ILD | p-value |
| Age at examination, years | 48 | 49.7 ± 12.4 | 22 | 54.5 ± 10.9 | 0.13^a^ |
| Female sex, n (%) | 48 | 36 (75) | 22 | 17 (77) | 0.84^b^ |
| Height, cm | 48 | 170 ± 9 | 22 | 170 ± 9 | 0.95^a^ |
| Weight, kg | 48 | 73.3 ± 16.5 | 22 | 73.9 ± 19.3 | 0.84^a^ |
| Body mass index, kg/m^2^ | 48 | 25.3 ± 4.7 | 22 | 25.6 ± 5.6 | 0.84^a^ |
| BP systolic, mmHg | 48 | 126 ± 18 | 22 | 123 ± 18 | 0.49^a^ |
| BP diastolic, mmHg | 48 | 73 ± 9 | 22 | 72 ± 10 | 0.60^a^ |
| HR, beats/min | 48 | 73 ± 17 | 22 | 74 ± 20 | 0.84^a^ |
| Smokers, daily, n (%) | 48 | 27 (56) | 22 | 9 (41) | 0.23^b^ |
| LDL cholesterol, mmol/l | 48 | 2.9 ± 0.9 | 20 | 3.1 ± 1.0 | 0.25^a^ |
| HDL cholesterol, mmol/l | 48 | 1.6 ± 0.4 | 20 | 1.3 ± 0.4 | 0.04^a^ |
| HDL/LDL ratio | 48 | 0.6 ± 0.3 | 20 | 0.5 ± 0.2 | 0.04^a^ |
| Total cholesterol, mmol/l | 48 | 4.7 ± 1.0 | 20 | 4.9 ± 1.1 | 0.40^a^ |
| NT-proBNP, ng/l | 47 | 15.2 ± 18.6 | 22 | 15.7 ± 14.4 | 0.92^a^ |
| Creatine kinase, U/l | 46 | 98 ± 71 | 22 | 140 ± 184 | 0.19^a^ |
| HbA1c, % | 47 | 5.6 ± 0.3 | 20 | 5.5 ± 0.6 | 0.76^a^ |
| CRP, mg/l | 46 | 3.1 ± 7.0 | 22 | 5.7 ± 10.7 | 0.24^a^ |
| ESR, mm/h | 48 | 11.7 ± 9.1 | 22 | 18.3 ± 18.4 | 0.05^a^ |
| Anti-RNP, median (IQR), x 10^-3^, U/l | 48 | 61.1 ± 80.2 | 22 | 125.4 ± 98.3 | 0.006^a^ |
| Current medication | | | | | |
| Ca^2+^ antagonists, n (%) | 48 | 8 (17) | 22 | 5 (23) | 0.55^b^ |
| β adrenoceptor blockers, n (%) | 48 | 3 (6) | 22 | 1 (5) | 0.78^b^ |
| Statins, n (%) | 48 | 3 (6) | 22 | 1 (5) | 0.78^b^ |
| Angiotensin-II- receptor blockers, n (%) | 48 | 1 (2) | 22 | 2 (9) | 0.18^b^ |
| Vitamin–K antagonists, n (%) | 48 | 0 (0) | 22 | 1 (5) | 0.14^b^ |
| COX-1 inhibitors, n (%) | 48 | 6 (13) | 22 | 2 (9) | 0.68^b^ |
| Nitrates, n (%) | 48 | 1 (2) | 22 | 0 (0) | 0.5^b^ |
| Diuretics, n (%) | 48 | 1 (2) | 22 | 0 (0) | 0.5^b^ |
| I_1_- Imidazoline receptor, n (%) | 48 | 0 (0) | 22 | 0 (0) | NA |
| P2Y-inhibitors, n (%) | 48 | 0 (0) | 22 | 0 (0) | NA |
| Values are mean ± SD. BP, blood pressure; Ca^2+^, calcium; COX-1, cyclooxygenase-1; HbA1c, glycated haemoglobin; HDL, high-density lipoprotein; HR, heart rate; LDL, low-density lipoprotein; NT-pro-BNP, N-terminal pro-BNP; ^a^ Student’s t-test; ^b^X^2^ test; | | | | | |

# Supplementary Table S4

| Supplementary Table S4. Electrocardiographic and echocardiographic characteristics in mixed connective tissue disease patients with and without interstitial lung disease at follow-up. | | | | | |
| --- | --- | --- | --- | --- | --- |
| Variables | n | Patients -ILD | n | Patients +ILD | p-value |
| *ECG* | | | | | |
| P, ms | 47 | 90 ± 16 | 22 | 88 ± 18 | 0.13^a^ |
| PR, ms | 47 | 163 ± 23.7 | 22 | 167 ± 26 | 0.36^a^ |
| QRS, ms | 48 | 85 ± 13 | 22 | 84 ± 24 | 0.22^a^ |
| QTc, ms | 48 | 400 ± 31 | 22 | 390 ± 33 | 0.02^a^ |
| Pathological ECG, n (%) | 48 | 9 (19) | 22 | 5 (23) | 0.70^b^ |
| Echocardiography |  |  |  |  |  |
| Fractional shortening, % | 48 | 37.8 ± 6.7 | 21 | 38.5 ± 6.4 | 0.70^a^ |
| MAPSE, mm | 47 | 13.4 ± 1.9 | 21 | 13.5 ± 2.0 | 0.81^a^ |
| TAPSE, mm | 45 | 22.7 ± 4.4 | 21 | 22.5 ± 3.4 | 0.85^a^ |
| MV E velocity, m/s | 48 | 0.73 ± 0.15 | 21 | 0.69 ± 0.16 | 0.32^a^ |
| MV A velocity, m/s | 47 | 0.59 ± 0.18 | 21 | 0.61 ± 0.17 | 0.58^a^ |
| MV E/A ratio | 47 | 1.31 ± 0.39 | 21 | 1.22 ± 0.35 | 0.31^a^ |
| TR V_max_, m/s | 48 | 1.67 ± 0.59 | 21 | 1.96 ± 0.46 | 0.05^a^ |
| sPAP, mmHg | 48 | 12.9 ± 7.8 | 21 | 16.4 ± 7.2 | 0.09^a^ |
| e’ velocity, m/s | 48 | 0.10 ± 0.02 | 21 | 0.09 ± 0.02 | 0.08^a^ |
| E/e’ ratio | 48 | 7.97 ± 2.39 | 21 | 8.43 ± 2.45 | 0.47^a^ |
| LV global longitudinal strain, % | 48 | 22.7 ± 2.6 | 20 | 21.7 ± 3.8 | 0.22^a^ |
| LV global circumferential strain, % | 46 | 27.4 ± 4.3 | 21 | 27.4 ± 4.3 | 0.97^a^ |
| RV basal diameter, cm | 42 | 3.4 ± 0.5 | 18 | 3.4 ± 0.6 | 0.95^a^ |
| RVOT proximal diameter, cm | 42 | 2.9 ± 0.4 | 17 | 3.0 ± 0.4 | 0.34^a^ |
| RV longitudinal diameter, cm | 35 | 6.9 ± 0.7 | 16 | 6.9 ± 0.8 | 0.99^a^ |
| RV systolic pressure, mmHg | 36 | 17.7 ± 8.2 | 17 | 22.5 ± 6.9 | 0.04^a^ |
| Values are mean ± SD. e’, early diastolic mitral annulus velocity; LV, left ventricle/ventricular; MAPSE, mitral annular plane systolic excursion; MV, mitral valve; MV A, MV A-wave; MV E, MV E-wave; P, P-wave; PR, PR interval; QRS, QRS complex; QTc, corrected QT Interval; RV, right ventricle/ventricular; sPAP, systolic pulmonary artery pressure; TAPSE, tricuspid annular plane systolic displacement; TR, tricuspid regurgitation; ^a^ Student’s t-test; ^b^X^2^ test | | | | | |

s
